# Genome sequence and global sequence variation map with 5.5 million SNPs in Chinese rhesus macaque

**DOI:** 10.1186/gb-2011-12-7-r63

**Published:** 2011-07-06

**Authors:** Xiaodong Fang, Yanfeng Zhang, Rui Zhang, Lixin Yang, Ming Li, Kaixiong Ye, Xiaosen Guo, Jun Wang, Bing Su

**Affiliations:** 1 Beijing Genomics Institute-Shenzhen, Chinese Academy of Sciences, Shenzhen 518083, China; 2 State Key Laboratory of Genetic Resources and Evolution, Kunming Institute of Zoology and Kunming Primate Research Center, Chinese Academy of Sciences, Kunming 650223, China; 3 Graduate School of Chinese Academy of Sciences, Beijing 100039, China; 4 Current address: Beijing Institute of Genomics, Chinese Academy of Sciences, Beijing 100029, China

## Abstract

**Background:**

Rhesus macaque (*Macaca mulatta*) is the most widely used nonhuman primate animal in biomedical research. A global map of genetic variations in rhesus macaque is valuable for both evolutionary and functional studies.

**Results:**

Using next-generation sequencing technology, we sequenced a Chinese rhesus macaque genome with 11.56-fold coverage. In total, 96% of the reference Indian macaque genome was covered by at least one read, and we identified 2.56 million homozygous and 2.94 million heterozygous SNPs. We also detected a total of 125,150 structural variations, of which 123,610 were deletions with a median length of 184 bp (ranging from 25 bp to 10 kb); 63% of these deletions were located in intergenic regions and 35% in intronic regions. We further annotated 5,187 and 962 nonsynonymous SNPs to the macaque orthologs of human disease and drug-target genes, respectively. Finally, we set up a genome-wide genetic variation database with the use of Gbrowse.

**Conclusions:**

Genome sequencing and construction of a global sequence variation map in Chinese rhesus macaque with the concomitant database provide applicable resources for evolutionary and biomedical research.

## Background

Rhesus macaque (*Macaca mulatta*) and human shared a most recent common ancestor about 25 million years ago [1] and their genome sequences share 93.5% identity [2]. Due to the genetic and physiologic similarity between rhesus macaque and human, rhesus macaques are the most widely used nonhuman primate animals for biomedical research, for example, in vaccine development and as animal models for human diseases [3-7]. In research, rhesus macaque subspecies from India and China are the most commonly used, and the divergence between these was estimated to be about 162,000 years ago [8]. The observed genetic divergence, though shallow, is considered to underlie the observed phenotypic differences between them, such as with regard to immune responses and disease progression. The well-known example is that, compared with Indian rhesus macaques, simian immunodeficiency virus (SIV) pathogenesis in Chinese rhesus macaques is closer to HIV-1 infections in untreated adult humans [9,10]. Although previous studies have determined thousands of SNPs and hundreds of microsatellite polymorphisms [8,11-16], a genome-wide high-density genetic variation map of rhesus macaque could provide much more comprehensive information. Therefore, developing a global map of genetic variations within and between Indian- and Chinese-derived rhesus macaques has important implications for biomedical research and drug development.

Here we sequenced the genome of a male Chinese macaque and compared the data with the released reference genome of an Indian macaque (rheMac2) [2]. We identified a total of 2.94 million SNPs that are heterozygous in the Chinese macaque and 2.56 million SNPs that are different between the Chinese macaque and the reference Indian macaque genomes. We also observed 123,610 deletions and other structural variations (SVs) by comparing Chinese with Indian macaques. We constructed a database, the Chinese Macaque Single Nucleotide Polymorphism (CMSNP) database, to display the SNPs and SVs using the Generic Genome Browser (GBrowse) platform. We have also integrated other valuable annotated information to enrich the CMSNP database, resulting in a comprehensive compilation of rhesus macaque genetic variations.

## Results and discussion

### Data generation

A peripheral blood sample was collected from a healthy male Chinese rhesus macaque; this was used for DNA extraction using the standard phenol/chloroform method. We performed whole-genome sequencing of this macaque genomic DNA sample using the Illumina Genome Analyser, with span sizes of the three paired-end DNA libraries ranging from 44 to 200 bp. In total, 33 gigabases of high quality sequences with 706.5 million reads (read lengths of 44 and 49 bp) were generated.

Using the improved Short Oligonucleotide Alignment Program (SOAP2) [17], we mapped the reads to the reference Indian macaque genome. A summary of the resequencing data is shown in Table 1 and in Table S1 in Additional file 1. In general, 92.64% of the reads can be mapped to a unique position in the reference genome and 95.95% of the bases in the reference genome are covered, resulting in an average 11.56-fold coverage. The relatively lower genome coverage in macaque resequencing compared with the more than 99% genome coverage in human resequencing [18,19] is likely due to the relatively low sequencing coverage of our study and the reference macaque genome assembly.

**Table 1:** Summary of the Chinese rhesus macaque re-sequencing data

Genome size	Effective length	Number of reads	Number of mapped reads	Number of bases	Number of mapped bases	Effective depth	Coverage (%)
2,864,106,071	2,646,668,809	706,459,956	654,493,989	33,064,980,424	30,607,016,223	11.56	95.95

### SNP identification

For SNP identification, we utilized a statistical model based on Bayesian algorithms that has been used in human resequencing analysis [18]. A consensus sequence (CNS) was then obtained, and a series of criteria were used to filter out the unreliable portion of the CNS for SNP detection (see Materials and methods). After filtering, a total of 5.5 million SNPs were detected (error rate ≤ 1%), of which 2.94 million are heterozygous (two alleles are different in Chinese macaque as supported by at least four reads for each allele; Figure S1a in Additional file 1). The remaining 2.56 million SNPs are homozygous (two alleles are the same in Chinese rhesus macaque but different from Indian macaque as supported by at least four reads; Figure S1b in Additional file 1). Assuming a Poisson distribution (λ = 11.56), the expected false discovery rate with four or more supporting reads is less than 0.001. It has been shown that the total number of SNPs would reach saturation at a sequencing depth greater than ten-fold using the paired-end reads [20]. Therefore, with 11.56-fold coverage, we likely uncovered all the SNPs in the genome of the Chinese macaque individual. The observed ratio of heterozygous to homozygous SNPs is 1.18, similar to the ratio observed for an individual human genome [21].

Compared with the previously identified 1,476 SNPs across five Encyclopedia of DNA Elements (ENCODE) regions in Chinese and Indian macaques (9 Chinese and 38 Indian rhesus macaques) [8], we completely identified 305 SNPs located in the same approximately 150-kb ENCODE regions, and 68.9% (210 of 305) of these are shared, indicating that most of the SNPs identified can be confirmed by the published dataset. Based on the shared SNPs, we conducted a hierarchical clustering analysis and the result indicates that the Chinese macaque sequenced in this study clusters with the Chinese macaques from [8] (approximately unbiased value is 94%; Figure S2 in Additional file 1), supporting the population identity of the sequenced Chinese macaque. Additionally, our results suggest that these SNPs could efficiently distinguish Indian-derived from Chinese- derived rhesus macaques [15].

**Figure 1:**
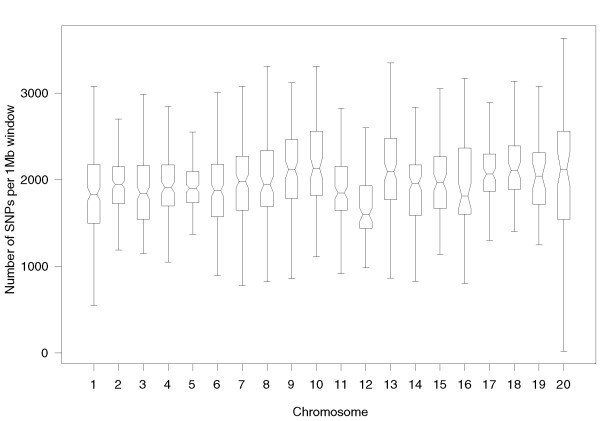
**Chromosomal distribution of the autosomal SNPs in 1-Mb windows**. Due to lower coverage (two-fold coverage), we have excluded the analysis of the sex chromosomes.

The chromosomal distribution of SNPs (excluding sexual chromosomes) per 1-Mb window is shown in Figure 1. The result indicates unbiased distribution of SNPs across the genome with a density of 2.08 SNPs per kilobase.

### Identification, verification and analysis of structural variation

**Figure 2:**
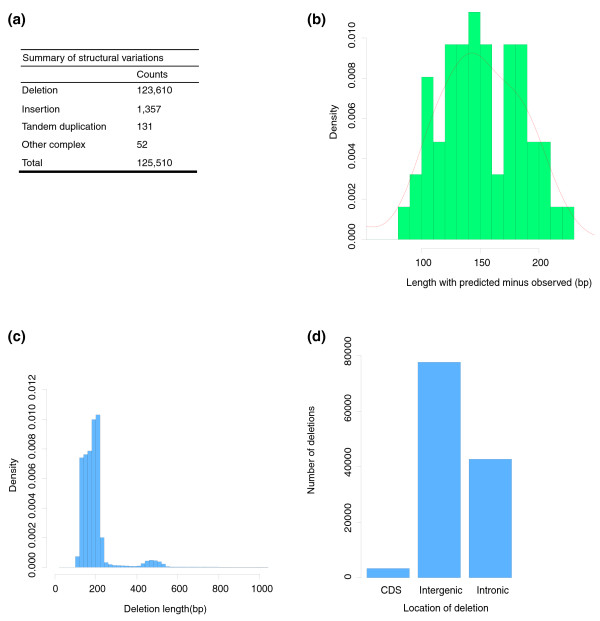
**Summary of the identified SVs**. **(a) **The type and number of SVs. **(b) **The size distribution of the predicted deletion lengths minus the observed deletion SV lengths for the experimentally tested deletion SVs. **(c) **The size distribution of the corrected deletion SVs. **(d) **The genome-wide distribution of deletions.

Paired-end sequencing is also a powerful tool for detecting genomic SV [22]. When reads are aligned onto the reference, a mated pair of reads should be in the correct orientation and the distance between them should be in an allowed range depending on the insert size of the sequenced library. If the mated pair of reads is not in a correct orientation or does not have an allowed span size, it may indicate a potential SV. We gathered abnormal mated pairs of reads for SV detection (see Materials and methods). After masking unreliable SVs located in gap regions of the reference genome (the sequence alignments crossing the gap regions), a total of 125,150 SVs were identified (Figure 2a), most of which (123,610, 98.8%) were deletions since deletions are easier to detect, consistent with previous reports [20,21,23]. There were 36,969 (30%) deletions overlapping with the repeat elements in the genome, and about half of the repeat elements (18,438) were Alu elements (Table S2 in Additional file 1).

To evaluate the reliability of the SVs based on our computational strategy, we focused on the deletions, which account for 98.8% of the identified SVs. We randomly selected 100 deletions (from the 123,610 candidate SVs) for PCR-based sequencing. Among them were 18 deletions located in repetitive regions in the genome that failed to be PCR amplified. The remaining 82 putative deletions were successfully amplified and sequenced; 74 of these are real deletions and the other 8 are false positive SVs (Table S3 in Additional file 2). Altogether, the deletion identification was highly accurate.

To further study the underlying mechanism of the deletions, we analyzed their length and sequence features. Based on the sequenced 74 deletions, we first compared the observed deletion lengths with the predicted pattern (Figure 2b). The length of the predicted deletion is 143 bp, on average, which is larger than that of the observed average (Table S4 in Additional file 2), likely due to the method used for identifying SVs (see Materials and methods). We further corrected the predicted deletion length and surveyed the size distribution of the deletions (Figure 2c). Genome-wide distribution of these deletions indicates that 62.8% of the deletions are located in intergenic regions and 34.5% are in intronic regions (Figure 2d). Compared with the randomly selected equivalent regions in the genome (a total of 105,000 regions with 5,000 from each of the 21 chromosomes), we observed a significant bias (*P *< 0.001, Chi-squared test with 1,000 replicates by Monte Carlo simulation) for intergenic regions, suggesting deletions occur more frequently in regions with low functional constraint.

We also tested whether the 74 experimentally verified deletions are polymorphic within Chinese macaque populations. We selected 20 Chinese rhesus macaques derived from four distinct geographical sites (5 from Sichuan Province, 5 from Yunnan Province, 5 from Guangxi Province, and 5 from Guizhou Province; Figure S3 in Additional file 1). Of the 74 deletions, 23 are fixed (homozygous), 45 are polymorphic, and the remaining 6 are uncertain (Table S5 in Additional file 2). This result suggests that a substantial portion of SVs inferred by comparing Chinese and Indian macaques are polymorphic, and those SVs fixed in Chinese macaques are particularly valuable as novel genetic markers for determining the geographic origins of macaques.

### Gene-based variations

Nonsynonymous SNPs are believed to make a significant contribution to phenotypic variation within populations [24]. They are also good candidate mutations that may explain Mendelian diseases. Thus, we mapped the 5.5 million SNPs to annotated macaque genes (Ensembl) [25] to identify the nonsynonymous SNPs in the genome; we found 43,959 SNPs in coding regions, of which 18,324 SNPs are nonsynonymous, accounting for approximately 41.7% of SNPs in the coding regions.

### Variations of orthologous disease and drug-target genes

One of the primary goals of the Chinese macaque genome resequencing is to maximize the use of the rhesus macaque genome sequence in the context of biomedical research. Revealing genetic variations located in disease-related and drug-target genes in macaques should be helpful to this purpose.

**Figure 3:**
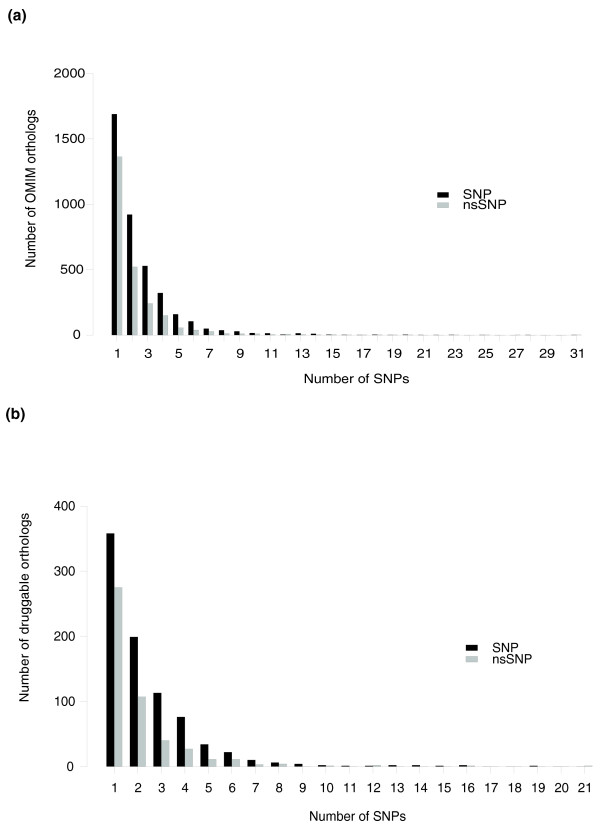
**Distribution of variants in the orthologous macaque disease and drug-target genes**. **(a) **The distribution of orthologous disease genes that contain one or more SNPs in their coding regions. **(b) **The distribution of the orthologous drug-target genes that contain one or more SNPs in their coding regions. SNP and nsSNP denote synonymous and non-synonymous SNPs, respectively. OMIM, Online Mendelian Inheritance in Man.

Our preliminary analysis identified a total of 6,823 macaque orthologs of human disease genes, of which 4,558 orthologs have at least one SNP in the coding regions, and 2,462 orthologs have at least one nonsynonymous SNP (Figure 3a). Overall, we observed 15,005 SNPs within the coding regions of these genes, of which 9,818 are synonymous and 5,187 are nonsynonymous. Additionally, we analyzed SV distribution in the 6,823 macaque disease orthologs. A total of 4,508 orthologous macaque disease genes bear at least one SV and there are 20,775 SVs within these genes (approximately 88.9% of SVs are located in introns).

We also performed a similar analysis on the drug-target genes. Genetic variants within the drug-target genes would have the potential to influence drug effects and could be a valuable resource for pharmacogenomic study. We mapped the variants to the macaque orthologs of human drug-target genes downloaded from DrugBank [26]. A total of 954 orthologous macaque drug-target genes have at least one SNP in the coding regions, and 483 of them bear at least one nonsynonymous SNPs (Figure 3B). Overall, we observed 2,980 SNPs within the coding regions of these genes, and 962 of them are nonsynonymous and 2,018 are synonymous. In addition, the 949 orthologous macaque drug-target genes have at least one SV in the genomic regions and there are 4,091 SVs within these genes.

Protein domains form functional units that are often the targets of drugs; these are called 'druggable domains' [27]. Thus, nonsynonymous SNPs within druggable domains are more likely correlated with clinical variations during drug treatment. To study this, we used 962 identified nonsynonymous SNPs within the coding regions of 483 macaque drug-target orthologs to identify SNPs within the druggable domains (see Materials and methods). A total of 478 nonsynonymous SNPs located in 273 unique genes were identified in the druggable domains (Table S6 in Additional file 3). Meanwhile, to detect whether these SNP-containing druggable domains in the macaque drug-target orthologs also have SNPs in their human counterparts, PolyDoms, a previously developed database that maps all coding SNPs in protein domains [28], was used to search for SNPs located in the same domains using macaque orthologs as the query. In total, 671 unique nonsynonymous SNPs were discovered in the same druggable domains (Table S6 in Additional file 3). These shared druggable domain SNPs between Chinese macaque and human provide a highly useful tool to access between-individual drug treatment variations in preclinical trials using macaques.

### The Chinese macaque genetic variation database

We have established the Chinese Macaque Single Nucleotide Polymorphism (CMSNP) database [29] for data visualization. We integrated our variation and other associated data into Gbrowse, a popular genome browser used in the GMOD project [30] (Table 2). We have also integrated annotated macaque genes [25] and microRNAs [31] into the CMSNP database for the purpose of understanding genetic variations at the gene level, as well as orthologous macaque disease and drug-target genes, which is helpful to further biomedical research. Finally, the evolutionarily conserved regions between human and macaque were added in Gbrowse [32], which can be used to understand the genetic variations within these conserved regions. All data have been organized into a MySQL relational database, which is efficient in retrieving data from indexed files.

**Table 2:** Datasets integrated into the CMSNP database

Datasets	Source
SNPs	Sequencing
SVs	Sequencing
Macaque genome sequence	NCBI database
OMIM orthologs	OMIM database [33]
Drug-target orthologs	DrugBank database [26]
Macaque annotated genes	Ensembl database (release 51) [25]
Macaque microRNAs	Sanger mirBase (release 12.0) [31]
Evolutionarily conserved elements	ECRBase [32]
Promoter dataset	ECRBase
Synteny dataset	ECRBase

OMIM, Online Mendelian Inheritance in Man.

**Figure 4:**
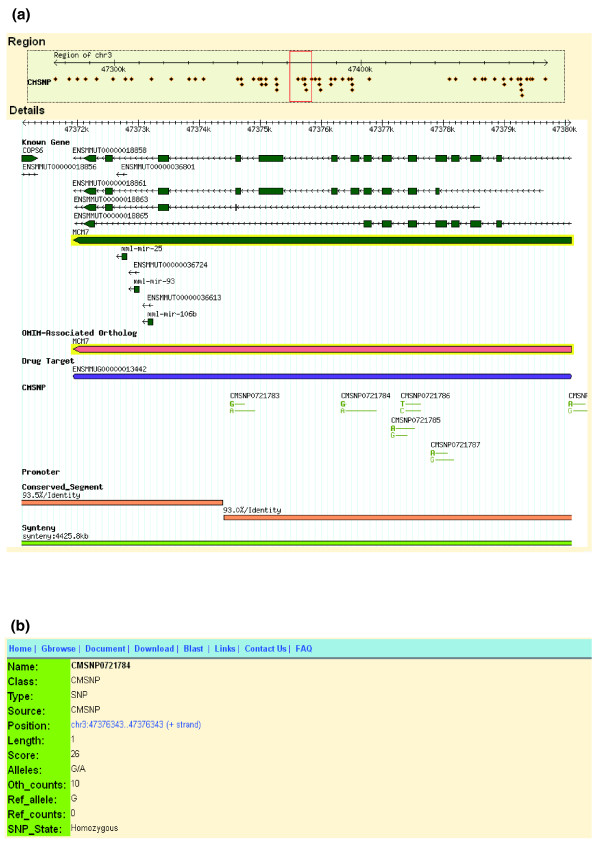
**Screenshots of the browser window of a specific region**. **(a) **The Region panel is an overview of SNP distribution in a specific region and SNPs are displayed as a glyph of triangles. The detailed panel shows the types and counts of SNPs, and the bold green-colored type is the reference allele. Other tracks, such as biomedical associations and conservation, are also displayed with glyphs and colors. **(b) **More detailed descriptions of each SNP are linked for viewing.

The CMSNP database is loaded in large batches and used primarily in read-only mode. An overview of the browser window is shown in Figure 4. The query forms supported in the CMSNP database include gene nomenclature, sequence coordinates, and CMSNP IDs, which are recorded by appending seven numbers (for example, *CMSNP0000001*). Individual entries within a track have either associated internal pages that provide information about the annotation or related links to external sites and databases.

## Conclusions

The variation map of rhesus macaque provides a useful framework for further genome-wide association studies and also has important applications to evolutionary and functional studies.

## Materials and methods

### DNA sequencing

Genomic DNA was purified from a 4-year-old male Chinese rhesus macaque from Sichuan Province of China. The standard phenol-chloroform method was used for DNA extraction.

The genomic DNA was fragmented by nebulization with compressed nitrogen gas. The overhangs of the fragments were converted to blunt ends using T4 DNA polymerase and Klenow polymerase. After adding an 'A' base to the blunt ends of the double-stranded DNA fragments, adaptors with 'T' base overhangs were ligated to the genomic DNA fragments. These fragments were separated on an agarose gel and excised from the gel at the DNA band around 200 bp. Finally, the DNA fragments were enriched by a ten-cycle PCR process.

DNA sequencing was performed using an Illumina Genome Analyser (Solexa, San Diego, California USA) according to the manufacturer's instructions. Fluorescent image deconvolution, quality value calculation and sequence conversion were carried out using the Illumina base-calling pipeline.

### SNP and structural variation identification

All sequenced reads were aligned to the reference rhesus macaque genome (rheMac2) using SOAP2 [17], with two mismatches allowed. After alignment, we used a statistical model based on Bayesian theory and the Illumina quality system to calculate the probability of each possible genotype of each position on the reference genome. At each position, the genotype was called by the highest probability, and a CNS was then obtained. The final CNS probabilities were transformed to quality scores in the Phred scale.

SNPs were obtained by a combination of parameters set to filter the CNS. The candidate SNPs were extracted from the CNS and then filtered using defined criteria to obtain the final SNP set. The filter criteria used included a Q20 quality cutoff (quality score ≥ 20 or error rate < 1%), estimated copy number of flanking sequences (< 2), minimum distance of two given SNPs (≥ 5 bp), and overall depth (≤ 100) in a given position in the reference. For both homozygous and heterozygous SNPs we required the support of at least four reads for each allele. Using cumulative Poisson statistics (λ = 11.56), the expected false discovery rate with four or more supported reads is less than 0.001. We compared the called SNPs in this study with previously identified SNPs across five ENCODE regions for data evaluation. Hierarchical clustering analysis with 1,000 bootstraps based on the locally shared SNPs was conducted to determine the population identity of the sequenced Chinese macaque. We also determined the chromosomal distribution of SNPs (excluding sexual chromosomes due to half coverage compared with autosomes) using 1-Mb windows.

According to the span size between the mapped paired-end reads and their orientations, alignments are divided into two types. The first type is the normal mated pair, which has the correct orientation and an allowed span size, and the other type is defined as an abnormal mated pair, which can be used for SV detection. SVs were called if the lengths were more than three times the standard deviation of the insert size of the DNA library. The insert sizes of all libraries constructed were 200 bp. For SV identification, we grouped the abnormal read pairs into diagnostic paired-end clusters. In order to avoid misalignment, each detected SV should be supported by at least four reads. We then examined and organized the SVs into alignment models, including deletion, insertion, inversion, translocation, duplication, and so on. Different types of SVs have a predefined mated pair alignment pattern that is inferred from the Solexa sequencing technology. For example, if there is a deletion in the sequenced individual, the mated pair of reads across the break point may have an abnormal span size but the correct orientation when aligned to the reference. SVs that overlap another SV in a spanned region were defined as complex SVs.

### Verification of structural variation

To verify SV, we randomly chose 100 deletions. Primers were designed by using the deletion region and the flanking 150-bp sequences (primers are listed Table S3 in Additional file 2). In addition, we also tested whether these deletions are polymorphic by screening 20 Chinese rhesus macaques from different geographic origins. All the 20 Chinese rhesus macaques were males and the blood samples were obtained from Kunming Primate Research Center, Chinese Academy of Sciences. DNA was isolated by the standard phenol-chloroform method.

### SNPs in disease genes

The identification of disease gene orthologs in the macaque genome was conducted through canonically reciprocal best-to-best hits implemented in the BLASTP program with default parameters between human proteins encoded by disease genes compiled from the Online Mendelian Inheritance in Man database [33] and macaque annotated proteins (for each gene, the longest transcript was selected); all synonymous and nonsynonymous SNPs were then annotated and assigned to macaque orthologs of human disease genes.

### SNPs in druggable protein domains

For identification of nonsynonymous SNPs within druggable protein domains, first all orthologous macaque druggable protein targets were identified through canonically reciprocal best-to-best hits implemented in the BLASTP program with default parameters between human druggable protein targets downloaded from the DrugBank database [26] and macaque annotated proteins (for each gene, the longest length of CDS was selected). A series of Perl scripts were then parsed to identify 483 nonsynonymous SNP-containing druggable orthologs. Finally, based on druggable human protein domains documented in the DrugBank database, human druggable protein targets were blatted against protein domain data downloaded from the Pfam (23.0) database [34] to identify the location of druggable domains; MUSCLE [35] alignment followed by Perl scripts were used to extract the corresponding nonsynonymous-SNP-containing druggable domains.

To identify the human SNPs within the domains detected in macaque, we used 273 gene symbols as queries to search in the PolyDoms database, which integrates all coding SNPs in human protein domains.

### Database construction

The CMSNP database contains two main datasets, an SNP dataset and an SV dataset, which are generated from our resequencing data. The annotated macaque gene dataset and the known macaque microRNA dataset were obtained from the Ensembl Biomart (release 51) [25] and the Sanger miRBase (release 12) [31], respectively. We also downloaded three additional datasets from the evolutionarily conserved regions database (ECRBase) [32], including the promoter dataset, the synteny dataset (between human and macaque), and the evolutionarily conserved region dataset.

A series of Perl scripts were used to convert these datasets into the GFF (General Feature Format) file format. Then, as the Gbrowse tutorial [36] recommends, we used bp_load_gff.pl to import all GFF-formatted files into the MySQL relational database.

### Data accessibility

The sequence data have been deposited in the NCBI Short Read Archive [37] under accession number [SRA037810].

## Abbreviations

bp: base pair; CMSNP: Chinese Macaque Single Nucleotide Polymorphism; CNS: consensus sequence; ENCODE: Encyclopedia of DNA Elements; PCR: polymerase chain reaction; SNP: single nucleotide polymorphism; SV: structural variation.

## Authors' contributions

BS and JW designed the study; XF, YZ, RZ, XG LY, ML and KY carried out sequencing and data analysis; XF, YZ, RZ, JW and BS wrote the manuscript. All authors read and approved the final manuscript.

## Supplementary Material

Additional file 1 **Tables S1 and S2 and Figures S1 to S3**. Table S1: detailed summary of Chinese rhesus macaque resequencing data. Table S2: summary of the overlapping deletions with repeat elements. Figure S1: cumulative density of read counts for homozygous and heterozygous SNPs. Figure S2: hierarchical clustering of rhesus macaques. Figure S3: distribution of the 20 Chinese rhesus macaques used for SV polymorphism testing. Click here for file

Additional file 2 **Supplementary tables**. Table S3: information for selected SVs and primers used for PCR and sequencing. Table S4: deletion length information. Table S5: SV status in Chinese macaque population. Click here for file

Additional file 3 **Table S6**. SNPs in drug-target protein domains. Click here for file
