# Supplementary material for: Genome sequence and global sequence variation map with 5.5 million SNPs in Chinese rhesus macaque
Source: Genome Biol. 2011 Jul 6;12(7):R63. doi: 10.1186/gb-2011-12-7-r63 (PMC3218825; doi:10.1186/gb-2011-12-7-r63)
Supplement: Additional file 1 — Tables S1 and S2 and Figures S1 to S3. Table S1: detailed summary of Chinese rhesus macaque resequencing data. Table S2: summary of the overlapping deletions with repeat elements. Figure S1: cumulative density of read counts for homozygous and heterozygous SNPs. Figure S2: hierarchical clustering of rhesus macaques. Figure S3: distribution of the 20 Chinese rhesus macaques used for SV polymorphism testing. [file gb-2011-12-7-r63-S1.DOC]

**Supplemental Table 1**. Detailed summary of the Chinese rhesus macaque resequencing data

| **Chr** | **Genome** | **Effective Genome** | **Coverage (%)** | **Block** | | **Depth** |
| --- | --- | --- | --- | --- | --- | --- |
|  |  |  |  | **Length** | **Rate (%)** |  |
| chr1 | 228,252,215 | 213,446,420 | 99.518 | 188,608,998 | 88.364 | 10.70 |
| chr2 | 189,746,636 | 181,477,283 | 99.534 | 161,562,609 | 89.026 | 10.22 |
| chr3 | 196,418,989 | 174,221,717 | 99.485 | 153,862,176 | 88.314 | 11.46 |
| chr4 | 167,655,696 | 160,215,117 | 99.468 | 142,102,331 | 88.695 | 10.49 |
| chr5 | 182,086,969 | 174,874,253 | 99.433 | 154,665,910 | 88.444 | 10.06 |
| chr6 | 178,205,221 | 170,378,098 | 99.513 | 150,636,162 | 88.413 | 11.90 |
| chr7 | 169,801,366 | 158,593,506 | 99.547 | 140,211,016 | 88.409 | 11.50 |
| chr8 | 147,794,981 | 137,021,754 | 99.525 | 121,432,627 | 88.623 | 11.57 |
| chr9 | 133,323,859 | 123,067,871 | 99.540 | 109,388,435 | 88.885 | 11.52 |
| chr10 | 94,855,758 | 86,818,744 | 99.557 | 76,783,985 | 88.442 | 11.69 |
| chr11 | 134,511,895 | 127,686,357 | 99.526 | 111,925,308 | 87.656 | 11.20 |
| chr12 | 106,505,843 | 101,395,563 | 99.555 | 90,524,896 | 89.279 | 11.27 |
| chr13 | 138,028,943 | 125,241,721 | 99.506 | 111,040,768 | 88.661 | 11.45 |
| chr14 | 133,002,572 | 120,216,370 | 99.528 | 106,587,194 | 88.663 | 13.47 |
| chr15 | 110,119,387 | 104,145,691 | 99.546 | 92,386,462 | 88.709 | 10.75 |
| chr16 | 78,773,432 | 70,457,785 | 99.490 | 60,552,208 | 85.941 | 11.22 |
| chr17 | 94,452,569 | 89,218,270 | 99.427 | 79,188,301 | 88.758 | 11.09 |
| chr18 | 73,567,989 | 69,717,142 | 99.507 | 62,484,397 | 89.626 | 10.45 |
| chr19 | 64,391,591 | 47,238,885 | 99.309 | 38,088,258 | 80.629 | 12.06 |
| chr20 | 88,221,753 | 69,329,831 | 99.405 | 35,517,335 | 51.230 | 11.26 |
| chrX | 153,947,521 | 141,500,845 | 32.964 | 60,911,194 | 43.047 | 1.91 |

**Supplemental Table 2**. Summary of the overlapping deletions with repeats. MIR, mammalian interspersed repeat; MLT, mammalian long terminal repeat; L1/L2, long terminal repeat; MER, repeat of endogenous retrovirus; others, simple repeats

| **Repeat elements** | **Number** |
| --- | --- |
| AluJ | 4,293 |
| AluY | 6,964 |
| AluS | 7,181 |
| MIR | 4,751 |
| MLT | 1,056 |
| L2 | 4,052 |
| L1 | 3,291 |
| MER | 2,345 |
| Others | 3,036 |
| Total | 36,969 |

**Supplemental Figure 1**. (A) The cumulative density of read counts for calling heterozygous SNPs, and (B) for calling homozygous SNPs. Ref_Allele refers to the allele in the reference Indian macaque. Other_Allele refers to the allele different from the reference Indian macaque.

**Supplemental Figure 2.** The hierarchical clustering of previously reported 47 rhesus macaques and the Chinese macaque in this study (termed CHSNP). Here, AU denotes for approximately unbiased (red), and BP for bootstrap probability (green). The AU p-value, which is computed by multi-scale bootstrap re-sampling, is a better approximation to unbiased p-value than BP value computed by normal bootstrap re-sampling.


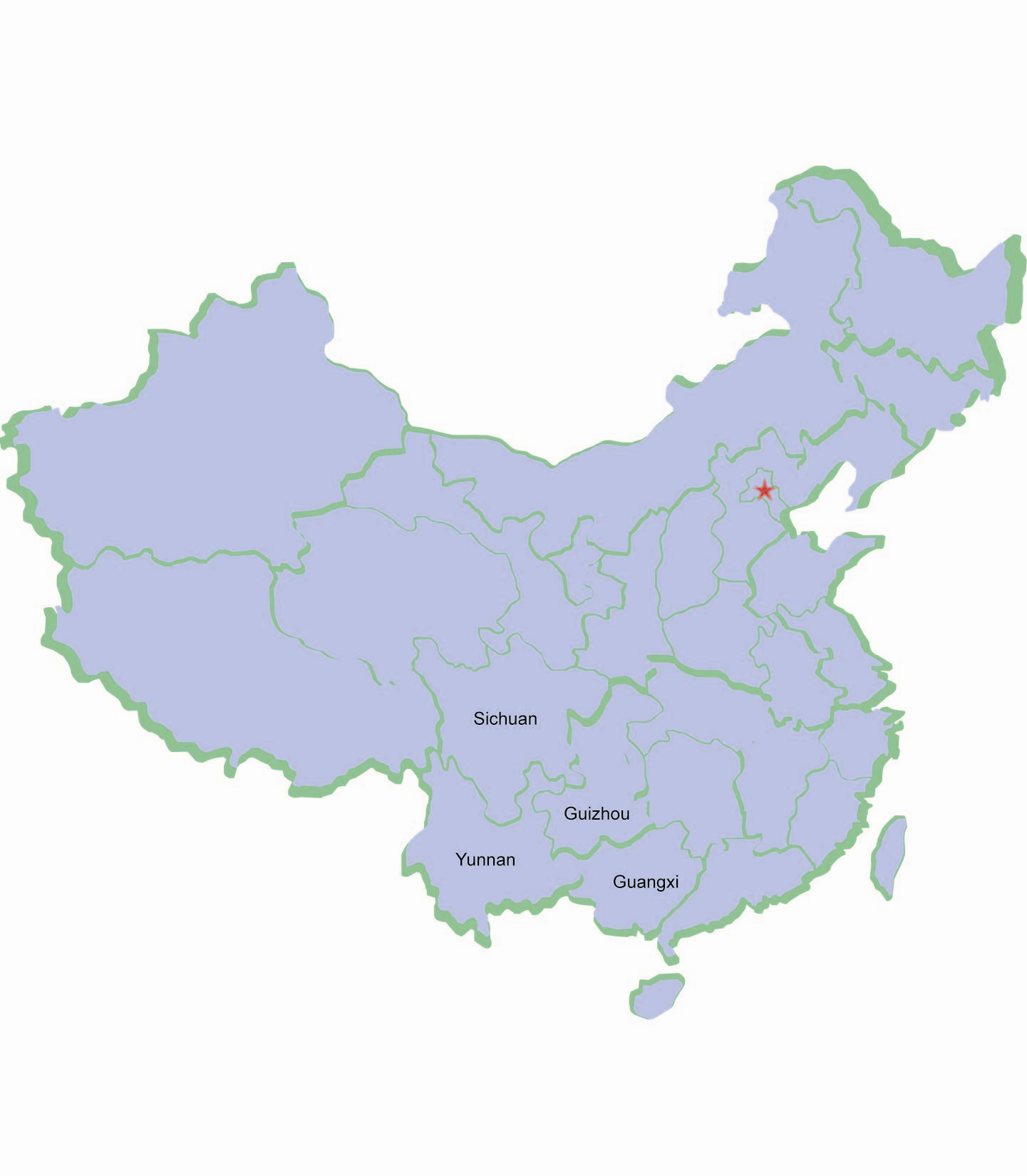


**Supplemental Figure 3**. The provincial distribution (Sichuan, Guizhou, Guangxi and Yunnan) of the 20 Chinese rhesus macaques used for SV polymorphism test.
